# Diverging Responses of Tropical Andean Biomes under Future Climate Conditions

**DOI:** 10.1371/journal.pone.0063634

**Published:** 2013-05-07

**Authors:** Carolina Tovar, Carlos Alberto Arnillas, Francisco Cuesta, Wouter Buytaert

**Affiliations:** 1 Centro de Datos para la Conservación, Universidad Nacional Agraria La Molina, Lima, Perú; 2 Long-term Ecology Laboratory, Biodiversity Institute, Department of Zoology, University of Oxford, Oxford, United Kingdom; 3 Consorcio para el Desarrollo Sostenible de la Ecorregion Andina, Quito, Ecuador; 4 Civil and Environmental Engineering, Imperial College London, London, United Kingdom; 5 Grantham Institute for Climate Change, Imperial College London, London, United Kingdom; George Washington University, United States of America

## Abstract

Observations and projections for mountain regions show a strong tendency towards upslope displacement of their biomes under future climate conditions. Because of their climatic and topographic heterogeneity, a more complex response is expected for biodiversity hotspots such as tropical mountain regions. This study analyzes potential changes in the distribution of biomes in the Tropical Andes and identifies target areas for conservation. Biome distribution models were developed using logistic regressions. These models were then coupled to an ensemble of 8 global climate models to project future distribution of the Andean biomes and their uncertainties. We analysed projected changes in extent and elevational range and identified regions most prone to change. Our results show a heterogeneous response to climate change. Although the wetter biomes exhibit an upslope displacement of both the upper and the lower boundaries as expected, most dry biomes tend to show downslope expansion. Despite important losses being projected for several biomes, projections suggest that between 74.8% and 83.1% of the current total Tropical Andes will remain stable, depending on the emission scenario and time horizon. Between 3.3% and 7.6% of the study area is projected to change, mostly towards an increase in vertical structure. For the remaining area (13.1%–17.4%), there is no agreement between model projections. These results challenge the common believe that climate change will lead to an upslope displacement of biome boundaries in mountain regions. Instead, our models project diverging responses, including downslope expansion and large areas projected to remain stable. Lastly, a significant part of the area expected to change is already affected by land use changes, which has important implications for management. This, and the inclusion of a comprehensive uncertainty analysis, will help to inform conservation strategies in the Tropical Andes, and to guide similar assessments for other tropical mountains.

## Introduction

Over the last decade, many studies have analyzed climate change impacts on biodiversity (e.g. [1], [2]). In mountain areas, one of the most important effects on biodiversity is the upslope migration of species [3], [4] or even entire biomes. The latter has been observed in many mountain regions, including Spain [5], [6], Alaska [7], the Swedish Scandes [8] and the Alps [9]. It is expected that these migrations will intensify in the future, highlighting the vulnerability of mountain biomes to climate change [10].

The Tropical Andes are a global biodiversity hotspot [11], and expected to be one of the most affected by climate change over the next 100 years [10], [12]–[14]. However, these projections have modelled biomes at relatively coarse resolutions (>50 km), which do not capture the heterogeneity of the Tropical Andes. Although studies with high resolution (5 km) exist for parts of the Tropical Andes, such as the Peruvian Yungas [15], no comprehensive study of climate change impact on biomes encompassing the entire Tropical Andes has been published. The Tropical Andes are not only important for their high levels of biodiversity [11], they also provide a wide range of ecosystem services, including water supply, carbon sequestration and fuel production [16]. Over 100 million people live in the Tropical Andes or in regions that depend directly on these natural resources [17]. Therefore, more detailed research is needed to understand climate change and its effects in this region.

Observations of historical climate trends [16], [18] indicate potentially very diverse changes in future climate. Some parts of the Andes such as the Bolivian highlands are expected to experience a reduced precipitation (−10%, with uncertainties of up to 50% point), and others such as the Ecuadorian and Peruvian highlands may see increases in precipitation ranging between 5% and over 60% [19]. The combination of a complex climate and topography with a highly diverse patchwork of biomes highlights the potential for very different and diverging responses to climate change in the Andes and different levels of vulnerability [20]. Indeed, for parts of the Andes a post-glacial upslope migration of biomes such as montane forest has been observed in response to warming [21], [22]. For other areas such as the Altiplano, the upslope migration of forest has stopped or even reversed due to a local response, for instance under influence of a microclimate such as that of the Titicaca Lake region [23].

This study analyses the potential impact of climate change in the biomes of the Tropical Andes. We aim to respond to two main scientific questions: 1) How will climate change affect the extent and elevational range of the Andean biomes? 2) Is it possible to identify regions most prone to change? Apart from the scientific insights, these results may help guide conservation strategies, by allowing conservation NGOs and government agencies responsible for ecosystem conservation to target biome areas that are most likely to persist under changing climate conditions.

Biome distribution models were developed to project the distribution of biomes under two future emission scenarios (A1B and A2) for two time slices (2010–2039 and 2040–2069). Given the high levels of uncertainty in future climate projections for the Andes [24] and the consequences of this for decision making [25], we used an ensemble approach to model future distribution of biomes.

Currently, land use already has a large effect on Andean biodiversity, which may either be reinforced or counteracted by climate change [26]. Therefore the outputs of the biome models were interpreted both as potential distribution and as remnant distribution, by disregarding for the latter any areas where vegetation is affected by human activities (denoted as human-modified areas).

## Materials and Methods

### 2.1 Study area

**Figure 1:**
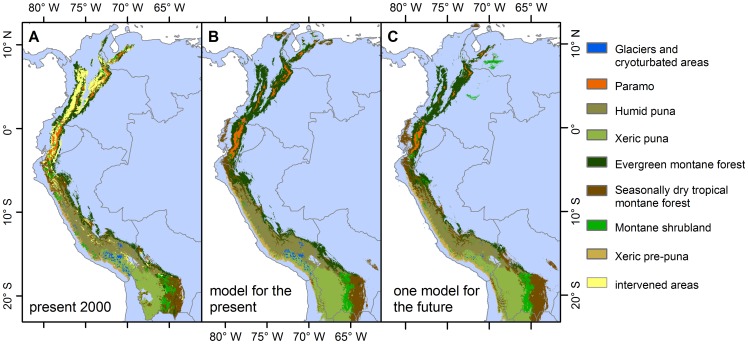
Biome maps. Current (observed) biome map (A) based on the Andean Ecological Systems Map [27], modelled potential biome map for the present 2000 (B) and an example of future biome map (C) using climatic variables of model gfdl_cm2_0 for A1B 2040–2069 scenario.

The Tropical Andes encompasses the Northern and Central Andes (Venezuela, Colombia, Ecuador, Peru and Bolivia) from 11°N to 23°S. The lower elevation limit is typically put at 600 m a.s.l. but this may vary according to the latitudinal location and mountain range [27]. The total area is around 1.27 million km^2^ (Table 1 and Figure 1A). Within the Tropical Andean region four major habitat types or biomes are found [28]: tropical and subtropical moist broadleaf forests; tropical and subtropical dry broadleaf forests; deserts and xeric shrublands; and montane grasslands and shrublands. However, given the importance of grasslands and shrublands in the highest part of the Tropical Andes, for example for conservation planning [29], vulnerability assessment [20], and ecosystem services [16]; we subdivided this biome into four categories (see Table 1). Therefore we defined seven Tropical Andean biomes: 1) paramo (P), 2) humid puna (HP), 3) xeric puna (XP), 4) evergreen montane forest (EMF), 5) seasonally dry tropical montane forest (SDTF), 6) montane shrubland (MS) and 7) xeric pre-puna (PP). Glaciers and cryoturbated areas (GC) were classified as a separate, eighth biome, to evaluate changes in the upper limit of the Andean region. The Tropical Andean biomes were obtained by grouping the ecological systems of the Andean Ecological Systems Map [27]. We used this map as the observed map (30 arc-seconds pixel size resolution, approximately 1 km in the equator) of the distribution of biomes for the year 2000 (Figure 1A). At the base of the Andes, the non-Andean biomes were defined as those that will possibly invade the Andean biomes under future climate change.

**Table 1:** Tropical Andean biomes, characteristic plant life-form and ordinal ranking based on humidity levels (from less humid to more humid) for each biome.

Biomes by Olson *et al.* [28]	Tropical Andean biomes	Area (%)	Plant Life-form	Humidity level
	glaciers and cryoturbated areas (GC)	1.5	desert	2
montane grasslands and shrublands	paramo (P)	3.2	grassland	5
	humid puna (HP)	18.6	grassland	4
	xeric puna (XP)	15.1	grassland	3
	montane shrubland (MS)	4.8	shrubland	6
tropical and subtropical moist broadleaf forests	evergreen montane forest (EMF)	19.3	forest	8
tropical and subtropical dry broadleaf forests	seasonally dry tropical montane forest (SDTF)	14.2	forest	7
deserts and xeric shrublands	xeric pre-puna (PP)	2.9	desert	1
Human-modified areas (human intervention)		20.5		
Total (1.27 million km^2^)		100.0		

### 2.2 Modelling approach

We modelled the potential distribution of each biome by using presence and absence points from the observed map as dependent variable and climatic and topographic variables as explanatory variables. Subsequently, we applied these models using future climatic variables to project future distribution of biomes. The outputs of eight climatic models were used to account for uncertainty. In addition to the present and future potential distributions we calculated remnant biome distributions which included human modified areas. Our approach is based on the following assumptions:

1. Current climatic conditions and the distribution of biomes are representative of climatic equilibrium conditions for the existing biomes. Every biome is modelled independently and each model represents the likeliness of occurrence of the existing biomes.
2. Future potential biome distributions should be interpreted as projected stabilised future biomes (in equilibrium with climate), and therefore conditional to the establishment of emerging areas. This process can take decades to centuries and is dependent, among other factors, on the rate of migration and establishment of representative species of each biome among other conditions, which are not studied here.
3. We used a static land use scenario for the distribution of remnant biomes. Although this does not allow taking into account future land-use dynamics, which would need separate land-use dynamics projections, it provides insights in the relative impact of respectively climate change and land use changes on Tropical mountain biota. This approach represents the lowest impact (optimistic) scenario due to climate change.

#### 2.2.1 Modelling potential biomes

Multiple backward stepwise logistic regression models were used to define the distribution for each Andean and non-Andean biome. The dependent variable (presence or absence of the biome) was obtained from the observed map. A subset of observations was used to construct the models. Points were sampled with a minimum distance of 4 pixels (approximately 4 km) in between to reduce spatial autocorrelation. Climatic and topographic characteristics were used as independent variables. We used initially the 19 bioclimatic variables from Worldclim [30] at 30 arc-seconds resolution (period 1950–2000) and two ombrothermic indexes [31] to represent the present conditions. A correlation matrix was constructed, and explanatory variables were selected such that a final set with minimal multicollinearity was obtained. These final explanatory variables were annual mean temperature, mean monthly temperature range, annual precipitation, precipitation of the driest month, precipitation seasonality calculated by the coefficient of variation, precipitation of the warmest quarter, precipitation of the coldest quarter, ombrothermic index and ombrothermic index of the driest bimonth. The latter two are based on the ratio of precipitation and temperature only in months with a positive temperature. We also included three topographic variables (Convergence index TCI, Terrain ruggedness index TRI and slope) as topography is an important factor influencing distributional patterns in the Andes [32]. These were calculated using a 30 arc-seconds resolution digital elevation model from the SRTM mission [33]. Some of the variables were log-transformed to obtain normality, and quadratic terms for all the variables were included to account for non-linear relationships (Table S1).

In this approach we obtained a probability map for each biome. To integrate all individual maps into a one final biome map for the present we overlaid all biome probability maps and selected for each pixel of the study area the biome with the highest probability of occurrence. As this procedure also assigns biomes to areas currently modified by human activities, it results in a potential biome map used as a baseline for the year 2000. Lastly, we calculated the 95% confidence interval of the probability of occurrence to analyse potential overlap with other projected biomes.

#### 2.2.2 Model validation

We used 4 indicators of model performance. First, for each biome model, a split sample test was applied, using 70% of the sampled points to calibrate the models and 30% for validation. The Akaike information criterion (AIC) was used as measure of fit. We evaluated each regression model through the ROC curve where values of the area under the curve (AUC) close to the unit indicate a good performance.

Next, using the 95% confidence interval for the predicted probabilities, we calculated for each pixel the number of non-selected biomes of which the confidence interval overlapped with the selected biome as a measure of biome model uncertainty.

As an over-all accuracy assessment, we compared the modelled baseline biome map (Figure 1B) with the observed biome map for 2000 (Figure 1A).

Lastly, to assess the risk of extrapolation beyond the model calibration envelope, we identified non-analogue future climate conditions, i.e., regions where values are outside the range of any variable used for calibration [34].

#### 2.2.3 Future potential biome maps

To obtain potential biome maps for the future, we ran the fitted biome distribution models using the future climatic conditions projected by the global climate models (GCMs) from the World Climate Research Programme's (WCRP's) Coupled Model Intercomparison Project phase 3 (CMIP3) multi-model dataset [35]. Climatic conditions were extracted for the periods 2010–2039 and 2040–2069, and for emission scenarios A1B and A2 using 8 models (bccr_bcm2_0, csiro_mk3_0, csiro_mk3_5, inmcm3_0, miroc3_2_medres, ncar_ccsm3_0, gfdl_cm2_0 and gfdl_cm2_1, using CMIP3 notation). These are all the CMIP3 models for which the climatic variables required for the biome modelling are available, i.e., monthly precipitation, daily minimum, mean and maximum temperature. Future climatic variables were obtained using the delta method on a monthly basis. We calculated the differences between future and present (anomalies or deltas) by subtracting the modelled simulations for the present of each variable and each month of the year from their correspondent values in the future. Afterwards the derived deltas were applied to observed temperatures and precipitation maps from Worldclim to obtain future climatologies at a finer resolution (30 arc-seconds resolution, approximately 1 km in the equator). The relative anomaly was used for precipitation, and the absolute anomalies for temperature (mean, minimum and maximum) [36].

#### 2.2.4 Remnant biomes

Areas with land cover affected by humans (i.e., human-modified areas) were extracted from the Andean Ecological Systems Map [27] and overlaid on to the potential biome maps for the present and the future climatic conditions to obtain the remnant biome maps.

### 2.3 Potential impact: Analysis of biome changes

#### 2.3.1 Changes in elevational range

A cumulative curve of the biome area as a function of elevation was plotted for each biome, emission scenario and period. The cumulative curve for present conditions and those for future climatic conditions were plotted together to identify significant shifts in elevation for each biome.

#### 2.3.2 Future changes in biome extents

Potential changes in biome extents were assessed using three measures: 1) areas that remained unchanged (stable areas), 2) emerging areas, where a biome is projected to occur in the future but not in the present and, 3) lost areas where a biome is likely to be replaced by another biome. For all three measures, the spread of the GCM model ensemble is summarised by reporting the minimum, median and maximum of the ensemble for each scenario and period. Both the potential and remnant biomes were analysed using this approach. To assess which biomes are projected to replace current biomes, a conversion matrix representing the percentage of change between the different biomes was calculated (only for the potential biome map). Also here the minimum, median, and maximum values of the GCM ensemble are reported.

#### 2.3.3 Regions most prone to biome change

We identified changes in areas between major physiognomy groups (desert, herbs/grasslands, shrubland, forest) and within them (levels of humidity, for example a projected change from xeric puna to humid puna) (Table 1), taking into account the agreement between the different models for each combination of scenario and period of time. In this approach any change between physiognomy groups will imply a change in vertical structure. If 80% of the biome models (at least 7 out of 8, each one using the outputs of the different climatic models) had a similar tendency, the area was assigned with one of the following categories: 1) increasing vertical structure, 2) either increasing vertical structure or increasing humidity level, 3) increasing humidity, stable plant physiognomy, 4) no change, 5) decreasing humidity, stable physiognomy, 6) either decreasing vertical structure or decreasing humidity level, 7) decreasing vertical structure. An eighth category was defined as inconsistency when less than 80% of the models agreed on the tendency of change.

## Results

### 3.1 Biome model validation and future climate

The AUC values of all regression models exceed 0.9, suggesting good individual model performance (Table S1). For the integrated biome map of the present, 90.3% of the study area shows no overlap of confidence intervals between the selected and any other biome (Figure S1A). Areas of overlap mostly occur for selected biomes with low probabilities and high standard errors (Figure S2). The comparison between the final integrated model and the observed map gives an overall accuracy of 89% (Table 2), suggesting a similarly good performance. Some biomes show higher commission and omission errors than others. The montane shrubland biome in particular appears mixed with the SDTF and in a lower degree with the EMF. To a lesser degree, some SDTF areas tend to be classified as EMF (Table 2).

**Table 2:** Accuracy assessment of the modelled potential biome map for the present (thousands of pixels).

Biome	GC	P	HP	XP	EMF	SDTF	MS	PP	non-andean	PC/Pred
GC	16.6	0	2.4	4.3	0	0	0	0	0	71%
P	0	40.5	0.5	0	6.0	0	0.1	0	0	86%
HP	1.0	0	263.5	11.7	5.0	2.0	0.7	0.5	0	93%
XP	2.5	0	20.5	209.1	0	1.6	2.4	0.6	0	88%
EMF	0	6.8	8.6	1.8	224.2	13.2	2.1	0.1	34.6	77%
SDTF	0	0.2	11.1	15.7	42.7	114.9	18.2	2.4	15.6	52%
MS	0	1.5	1.9	1.4	9.8	20.9	31.3	5.9	2.1	42%
PP	0	0	1.0	1.4	0	2.6	1.6	36.3	1.2	82%
non-andean	0	0	0	0	22.3	6.2	0.1	1.2	1596.2	98%
PC/Obs	82%	83%	85%	85%	72%	71%	55%	77%	97%	89%

Rows represent the observed map (see methods) while columns represent the predicted biome for the present 2000. The number of pixels correctly identified by the model is shown in the diagonal values. PC/Obs: percentage of pixels correctly classified, PC/Pred: percentage of pixels correctly identified by the model. GC = glaciers and cryoturbated areas, P = paramo, HP = humid puna, XP = xeric puna, EMF = evergreen montane forest, SDTF = seasonally dry tropical montane forest, MS = montane shrubland, PP = xeric pre-puna.

The climate model ensemble projects, on average for the entire region, an increase in temperature between 1 and 1.5°C for 2010–2039 and between 2 and 2.5°C for 2040–2069 under the A1B scenario. The A2 scenario projects a further increase of around 0.5° on top of the previous figures. These projections are spatially homogeneous. On the contrary, precipitation predictions are much more variable. Generally, less than 7 of 8 climatic models agree on the direction of change. Since temperature patterns for the Andes are much better characterised than precipitation patterns [37], there may be an inherent bias in the biome models to fit better to temperature maps than to precipitation maps. An example of future biome distribution is shown in Figure 1C.

Lastly, non-analogue future climatic conditions (i.e., outside the range of calibrated data for each variable) are observed mostly for the non-Andean biomes, mainly in the north coast of Colombia for all scenarios and periods (Figure S1B as an example). Non-analogue climates are absent in the Andean region for the period 2010–2039, while for 2040–2069 they represent 0.02% (A1B) and 0.05% (A2) of the Andean region.

### 3.2 Changes in elevational range

**Figure 2:**
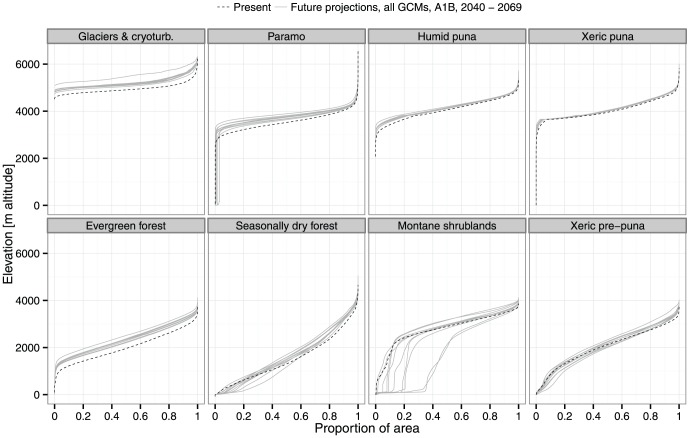
Elevational range changes for A1B 2040–2069. Glaciers and cryoturbated areas, paramo, humid puna and evergreen montane forest show upward displacement of the lower boundary. This can be observed in the left hand side of the accumulation curves, where curves of all models for the future (in grey) are higher than the curves for the present (dotted line). Seasonally dry tropical montane forest, montane shrubland and xeric pre-puna show downslope expansion in the lower boundary where future curves are lower than the present one. Upper boundary show upward displacement for almost all biomes, observed at the right hand side of the accumulation curves. The x-values were scaled from 0 to 1 to compare landscapes of different size.

The upper boundaries of almost all biomes show an upslope displacement (Figure 2). The only exceptions are the biomes restricted to the upper parts of the Andes, i.e. glaciers and cryoturbated areas, and the paramo. The trends for the lower limit of the distribution of each biome, however, are more variable. The majority of biomes are also projected to experience an upslope displacement of their lower limit (Figure 2). This shift is more marked for glaciers and cryoturbated areas, paramo, humid puna and the evergreen montane forest and to a lesser degree for the xeric puna. Yet our model projects downslope expansion of the lower boundary of several biomes: seasonally dry tropical montane forest, xeric pre-puna and especially montane shrubland. The puna biomes, and especially the xeric puna, show the least change in their elevational range.

### 3.3 Projected impacts of climate change in the extent of Andean biomes

**Figure 3:**
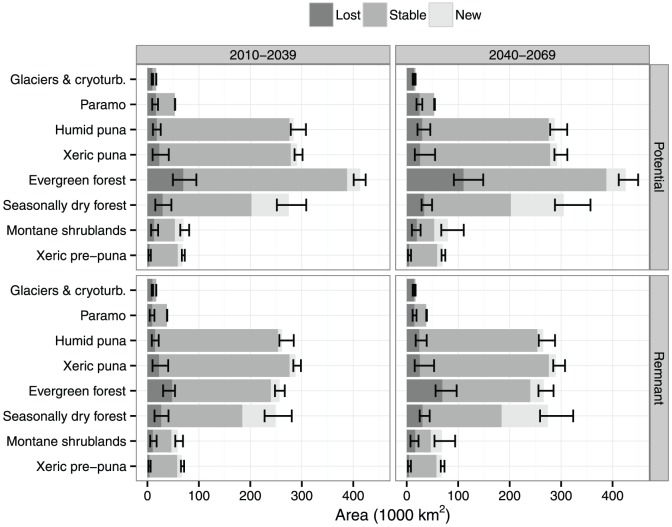
Median change in the area of potential biomes versus remnant biomes for A1B scenario period 2010–2039 and 2040–2069. In dark grey the lost areas (the biome will be replaced by another biome), in grey stable areas (areas that remained unchanged) and in light grey new or emerging areas (the biome is projected to occur in the future but not in the present). Black lines represent the minimum and maximum values of all models. The sum of the stable and lost areas represent the present area, while the sum of the stable and emerging areas represent the future projected area.

Future climate change will lead to a small general decrease of the area currently occupied by Andean biomes [sensu 27] according to the majority of the models, for both periods 2010–2039 (median of all models: A1B = −2.6%, A2 = −2.6%) and 2040–2069 (median of all models: A1B = −4.6%, A2 = −1.3%). For each case, only 1 or 2 models out of 8 project a small increase in the total area of Andean biomes. Despite the general decreasing trend, the magnitude of the projected changes varies across biomes. Our discussion concentrates on the minimum, median and maximum values of projected stable, lost and emerging biome areas (Figure 3 and Table S2) to characterize the uncertainty in the GCM model ensemble. For the potential biome map, the paramo glaciers and cryoturbated areas are expected to suffer the largest relative area loss in both emission scenarios, both periods and in all GCM models (**Figure 3** and Figure S3). For example, for the scenario A1B period 2010–2039, the glaciers and cryoturbated areas are projected to lose 57.7% of their current extent (median of all models, Table S2), mostly in favour of the expansion of xeric puna (Table 3). The lower end of the projection range still amounts to a loss of 49% (Table S2). Similarly, the projected median reduction in the extent of the high-altitudinal paramo grasslands is 31.4%, mostly to be replaced by EMF (Table 3). All models consistently project a net loss of paramo area (Figure S4, Figure S5, Figure S6 and Figure S7). Further in the future and under more severe emission scenarios, projected reductions are larger (Table S2).

**Table 3:** Conversion matrix of biomes from present to future.

Present/Future	GC	P	HP	XP	EMF	SDTF	MS	PP	NAB
GC	42.3	0.0	21.6	35.4	0.0	0.0	0.0	0.0	0.0
	(19.8–51)	(0–0)	(11.2–28.7)	(20.5–59.8)	(0–0)	(0–0)	(0–0)	(0–0)	(0–0.9)
P	0.0	68.6	0.5	0.0	25.4	0.1	1.5	0.0	1.1
	(0–0)	(57.4–82.4)	(0–4.7)	(0–0)	(12.5–39.4)	(0–0.3)	(1.1–1.9)	(0–0)	(0–7.9)
HP	0.0	0.1	93.4	1.7	3.4	1.3	0.4	0.1	0.0
	(0–0)	(0–0.2)	(88.9–96.2)	(0–4.2)	(2–3.8)	(0.3–3.5)	(0.1–1.3)	(0–0.4)	(0–0)
XP	0.0	0.0	1.2	91.7	0.0	3.9	0.7	1.2	0.3
	(0–0)	(0–0)	(0.1–8.5)	(85.1–96.4)	(0–0)	(2.1–6.4)	(0.2–2.3)	(0.9–1.8)	(0.2–0.6)
EMF	0.0	0.0	0.0	0.0	82.0	4.4	0.7	0.0	11.4
	(0–0)	(0–0.1)	(0–0)	(0–0)	(73.2–87.2)	(2.6–9.8)	(0.3–1.1)	(0–0)	(7.5–21.7)
SDTF	0.0	0.0	0.0	0.9	0.7	85.3	1.3	0.5	10.9
	(0–0)	(0–0)	(0–0.2)	(0.1–1.5)	(0.1–3.3)	(77–92.4)	(0.7–3.1)	(0.2–1.1)	(5.1–15.8)
MS	0.0	0.0	0.0	0.0	0.5	20.2	75.7	0.5	4.1
	(0–0)	(0–0)	(0–0.3)	(0–0)	(0.1–2.1)	(8.3–29.8)	(61.3–86.6)	(0–1.9)	(2.6–6.6)
PP	0.0	0.0	0.0	0.6	0.0	1.6	1.0	92.9	2.7
	(0–0)	(0–0.1)	(0–0.6)	(0–1.2)	(0–0)	(0.5–3.5)	(0–5)	(88.8–96.7)	(1.7–4.4)
NAB	0.0	0.0	0.0	0.0	0.1	1.4	0.2	0.2	98.1
	(0–0)	(0–0)	(0–0)	(0–0)	(0–0.3)	(0.7–2.2)	(0–0.6)	(0.1–0.4)	(96.6–98.7)

Median change in area (%) of all models, for scenario A1B 2010–2039, between potential present biomes (rows) and potential future biomes (columns). Minimum and maximum values of all models are shown in parentheses. GC = glaciers and cryoturbated areas, P = paramo, HP = humid puna, XP = xeric puna, EMF = evergreen montane forest, SDTF = seasonally dry tropical montane forest, MS = montane shrubland, PP = xeric pre-puna, NAB = non-andean biomes.

The EMF will suffer the largest absolute area loss for both scenarios and periods. The range of models projects higher areas of biome loss than emerging areas (Figure **3** and Figure S3). Around 69000 km^2^ (median, A1B, 2010–2039) of EMF is set to be replaced, mostly by non-Andean biomes and SDTF (Table 3 and Table S3). However, a significant part of this loss may be compensated by the expansion of EMF into other biomes (25400 km^2^, A1B, 2010–2039), mostly into areas that currently host paramo (Table 3 and Table S3).

The xeric and humid punas are expected to undergo both small losses and small gains, which offset each other largely and generate only a small impact in the total area of the potential biome map. Again, the projected area loss is slightly higher for 2040–2069 than for 2010–2039 (**Figure 3** and Figure S3).

Contrastingly, xeric biomes (xeric pre-puna and SDTF) may show an increase of their total current area because of a larger share of emerging areas compared to the losses (**Figure 3** and Figure S3). This is particularly conspicuous for the SDTF, which is projected to replace areas of predominantly montane shrubland and EMF (Table 3). During the period 2040–2069, this expansion is more prominent (Figure S3).

With the exception of glaciers and cryoturbated areas, the remnant area of all biomes is necessarily smaller than that of their potential distribution (**Figure 3**). The stable area of EMF in particular shows clearly that human-modified areas have already encroached a large part of the potential distribution of this biome, particularly in the Northern Andes (i.e. Colombia and Ecuador) (Figure 1A). Similarly, human-modified areas currently already occupy around half of the projected potential emerging areas of EMF.

However, when future changes are expressed relative to the current area, the differences between potential and remnant biomes are small for all biomes except for the paramo and montane shrubland (Table S2). For the paramo, a median loss of 31.4% is projected for the potential distribution, but this is only 25% for the remnant areas (A1B, 2010–2039). This pattern is consistent for all GCM models, ranging from a potential (remnant) loss of 38.6% (35.6%) for bccr_bcm2_0 to 17.3% (11.19%) for miroc3_2_medres. This observation suggests that climate change will mostly affect areas that are currently already affected by human activities. On the contrary, for biomes where the differences are small, it may suggest that climate change will have an equal impact on the natural and perturbed areas.

### 3.4 Regions most prone to biome change

**Figure 4:**
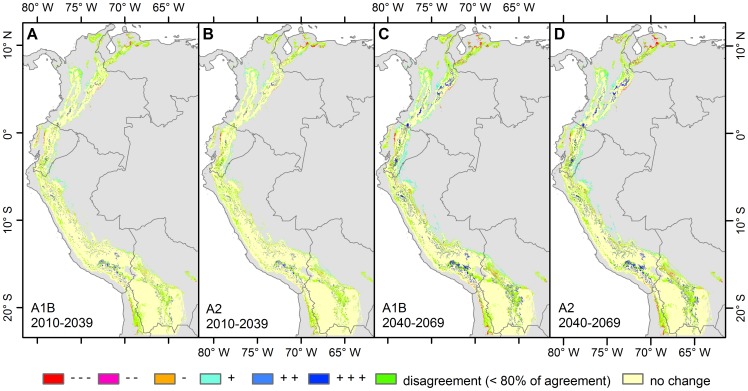
Agreement on the direction of the projected change between biome models using different climatic models. Calculations were made for scenario A1B 2010–2039 (A), A2 2010–2039 (B), A1B 2040–2069 (C) and A2 2040–2069 (D) based on physiognomy (desert, grassland, shrubland, forest) or humidity level. +++ Increasing vertical structure, ++ Either increasing vertical structure or humidity level, + Increasing humidity level, stable physiognomy, - Decreasing humidity level, stable physiognomy, -- Either decreasing vertical structure or humidity level, --- Decreasing vertical structure. Areas where less than 7 models agree on the direction of change are considered under the class “disagreement”.

For the scenario A1B and period 2010–2039, in 83.1% of the total area currently occupied by Andean biomes (potential modelled map), 7 or more models project that it will remain stable and no change in biome is projected (Table 4 and Figure 4). A similar value is reported for the A2 2010–2039 scenario, though these figures are lower for the period 2040–2069. In only 3.3% (scenario A2, period 2010–2039) or 3.8% (scenario A1B, 2010–2039) of the total study area, 7 or more models effectively project a change in biome. These figures increase for period 2040–2069 to 7.6% and 7.9% for scenario A2 and A1B respectively. In the remaining areas, less than 7 out of 8 models agree on the occurrence of change (Table 4 and Figure 4).

**Table 4:** Percentage of the present Andean area where more than 80% of the models (at least 7) agree on the direction of the change in physiognomy (desert, shrubland, grassland, forest) and/or humidity levels.

	A1B		A2	
	2010–2039	2040–2069	2010–2039	2040–2069
Decreasing vertical structure	0.1	0.3	0.2	0.3
Either decreasing vertical structure or humidity level	0.1	0.3	0.1	0.3
Decreasing humidity level, stable physiognomy	0.4	0.9	0.4	0.8
No change	83.1	74.8	83.1	75.0
Increasing humidity level, stable physiognomy	0.5	1.4	0.4	1.2
Either increasing vertical structure or humidity level	0.0	0.0	0.0	0.0
Increasing vertical structure	2.6	5.1	2.2	5.0
Inconsistency (areas with disagreement)	13.1	17.2	13.5	17.4

Areas of major change are located mainly in the ecotones (Figure 4). Such changes can be identified, for example, for the A2 scenario, for which 2.2% of the current Andean area is expected to experience an increase in vertical structure for the period 2010–2039 (Table 4) with higher values (5%) for 2040–2069. Areas following this pattern are the Boyaca paramo (Colombia), the paramo of Azuay and Loja (Ecuador), and the paramo of Piura and south Cajamarca (Peru). New projected climatic conditions are typically those of evergreen montane forest. Glaciers and cryoturbated areas are expected to follow the same trend especially in the region of Arequipa in South Peru and Central Ecuador. These areas are projected to be colonized by xeric or humid puna. Finally, 0.2% of the current Andean landscape would convert into a simpler vertical structure or into a biome with less humidity for scenario A2, period 2010–2039 (Table 4). This is most notable in the montane forest of the Eastern Cordillera in the province of La Paz (Bolivia), the montane forest of the department of San Martin (Peru), and on the Western versant of northern Ecuador (Pichincha and Cotopaxi provinces) (Figure 4).

## Discussion

It is expected that Andean biomes have different degrees of vulnerability to climate change (e.g. [20]). Our results indeed confirm that specific biomes are projected to be more affected than others in terms of reduction of their extent and shifts in elevational range. Additionally, our method allowed identifying those regions that are likely most prone to changes at a fine spatial resolution (1 km), while accounting for the inevitable uncertainties of climate projections. In the next sections, we discuss the projected changes and the implications for conservation of the biomes and regions most prone to change. Lastly, we briefly discuss the potential caveats of our modelling approach and the potential for future improvements.

### 4.1 Changes in Andean biomes

Our results project that most biomes will experience upslope displacement of the upper boundary, which implies a gradual replacement of one biome by another. However, the question remains how likely such a replacement is within the velocity of climate change in Andean biomes. Although upslope displacement has been observed for forest, paramo and punas in post-glacial times [21], [22], [38], [39] it is uncertain whether the right conditions for displacement are met under current climate change. For instance, temperature is now increasing at a faster rate than in post-glacial times [21], [40], which implies that biome displacement will require species to migrate faster. If this does not occur, many Andean species populations are likely to decline [26] and novel species assemblages likely to emerge. Nevertheless it is important to note that our approach is based on biome modelling and not on species distributions. Even though species composition might change, the vegetation physiognomy is the main characteristic that defines a biome. The establishment of the biome in potential emerging areas is a process that can take decades. Not only representative species of each biome will have to establish but also functional species or nurse plants that may act as facilitators of the colonization process [41]. Additionally, even though some individuals might be able to migrate, the establishment as stabilized biome (in equilibrium with climate) will require populations to adequately develop pollination and dispersal processes to assure reproduction. Migrating species will have also to face competition with currently existent species. If new climatic conditions are variable enough to encompass previous climatic conditions, competition would be stronger and migrating species would have more difficulties to establish [39].

Despite the abovementioned conditions, the upslope displacement of some biomes as a response to climate change has been observed in European mountains for the last 50 years [5], [6], [9]. This supports our projections of upslope displacement of the upper boundary of most biomes. For the Andean forest biomes, the limited carbon assimilation rates at higher elevations due to low night time temperatures [42] might be overcome by a temperature increase induced by climate change. In fact, present-day climate-driven migrations have already been recently reported for some tree species in the Andean region [4]. However, the rate of migration is lees than expected from the observed changes in temperature. In addition, limitations due to high radiation [43], soil types and humidity [16] will still be present. On the other hand, an elevation gradient in biotic interactions may act as positive force allowing upslope migration of trees in the Andes. A study suggests for instance that tree seed predation is lower at higher elevation [44].

A second important difference between post-glacial times and the present is that human influence is larger now than in the past. Andean landscapes nowadays are heavily transformed, for example the Central and Eastern mountain chain in Colombia [45], [46]. This affects potential emerging areas of some biomes, such as EMF (Figure 3 and Figure S3), and reduces their resilience. Current agriculture, grazing and burning practices in the border paramo/EMF and puna/EMF have already degraded many of these natural areas [26], [43], [47]. These practices have a strong influence on the present-day upper forest line on the Andes, and are likely also to have a critical role in controlling EMF upslope displacement under climate change scenarios [48], [49]. Upslope migration of the upper boundary is not only constrained by land use change but also by habitat fragmentation [50], [51], which might especially affect microrefugial expansion. This process is suggested as an important strategy in the Andes, based on the observation that only some populations of each species migrate while others collapse [39].

Dry biomes (SDTF, montane shrublands and xeric pre-puna) are the only biomes which lower boundaries are projected to expand downslope, suggesting a heterogeneous response within the Andes under climate change conditions. Historical evidence has started to appear showing such a downslope expansion for some plants [52], [53]. The main driver for this process seems to be a change in the climatic water balance [52]. The impact of a change in water availability may indeed supersede or interact with changes in temperature, hence leading to a more complex and strongly biome-specific response. In our case, the downslope expanding biomes are all dry biomes. This suggests that the temperature increase puts more pressure on the water availability (through an increasing evapotranspiration), which favours the downslope expansion of more drought-resistant biomes. However further studies are needed to explain this pattern in the Tropical Andes.

Interestingly, our projections at biome level indicate that most of the Andean area will remain within the same biome in contrast to what is predicted for species [4], [26]. Since biome models are mainly focused on physiognomic characteristics and not species composition it is likely that a biome model can encompass wider climatic characteristics than those for specific species. For example, an herbaceous species typical of montane forest may migrate to grassland biomes without causing a major change in biome. Species and biome modelling are complementary approaches [54] and future research in the Andes should focus on the integration of both. However, neither approach includes evolutionary processes and species plasticity. Hence they do not account for the possibility that species may adapt to new climatic conditions rather than to migrate [55]–[57].

### 4.2 Most affected biomes and regions: implications for conservation

While global projections suggest the Tropical Andes are among the most vulnerable areas under climate change [10], [12], [14] we find diverse responses among biomes and regions for the projected scenarios. The paramo grasslands and the glaciers and cryoturbated areas, located at the highest elevation, are most at risk due to the lack of upslope area for migration. They are projected to lose more than 30% of their present day area. Biomes located at mid-elevations have potentially more area to migrate towards. The steeper elevational gradient may allow them to reach their optima temperature at smaller distances than lowland biomes [58], [59]. Indeed, both montane forest biomes (EMF and SDTF) show an upslope displacement of both their upper and lower boundaries in the future projections, but only EMF would suffer a reduction of its total area. The projected replacement of EMF by lowland non-Andean biomes is one reason for this behaviour, as has been observed in the Holocene [21]. Another reason for the projected reduction of EMF is its replacement by dry forest taxa (SDTF). However this has not been observed during the Holocene. This is probably due to an alternation between dry and wet events [39], rather than a continuous dry period as modelled in our future climate projections (interannual climate variability was not included). It is uncertain whether such replacement by SDTF will occur in the future and information is still scarce to elucidate the ecological patterns of SDTF under climate change.

Land use changes complicate the situation for the most threatened biomes. Under the potential distribution scenario, part of the paramo grasslands is projected to be replaced by forest biomes. This is compatible with projections for other alpine grasslands in the world [7], [60], [61] and with paleo records of historical temperature increase [22]. In reality however, agricultural activities have already encroached parts of the paramo and forest, including the potential emerging areas of EMF (Figure 3 and Figure S3). Socioeconomic factors may drive this encroachment at present (e.g. [62]), but it is likely that climate change will contribute to the current expansion of agricultural areas by providing more suitable climates in upper areas [63]. Our approach should be considered as the baseline scenario (i.e., most optimistic) of climate change, where land use will stay the same. Under this approach the paramo grassland seems to be more affected by land use change than by climate change (Table S2), though an overall loss is projected for both potential and remnant scenarios (Figure 3 and Figure S3). Given that it is very likely land use change will increase in the future, the threat posed to this biome is even higher than to any other biome.

Potential changes into biomes with different physiognomy or different degrees of humidity would not only have ecological consequences but also would impact directly ecosystem services provided by the original biomes. In the case of a reduction in vertical structure, aboveground carbon storage will be reduced. Nevertheless, non-forested biomes such as the paramo, which have a simpler vertical structure, tend to have a larger belowground and soil carbon stock. Hence, the impact of any replacement of the paramo biome on the overall carbon storage may not be straightforward. On the other hand, areas with increasing humidity levels will be more susceptible, for example, to leaching processes until the vegetation stabilizes.

Although the identification of areas where most of the models agree in changes is useful for conservation management, the uncertainty in these projections remains problematic. Therefore, areas with no projected change or with a consistent change would be obvious target for conservation compared to those with large uncertainty. Additionally, fostering landscape networks (protected areas, connecting zones and intermediate landscapes) would be a more effective conservation strategy than isolated protected areas [64].

From this perspective, conservation strategies should be designed to fulfil at least three main criteria: (1) Conservation areas should ideally cover a large vertical range to capture the projected biome displacement as a way to maintain connectivity and ensure the integrity of functional processes such as water and carbon cycle and species migration. (2) Conservation strategies should include not just pristine habitats but also secondary forest and abandoned agricultural areas in the paramos and punas to promote restoration schemes to reduce land use change and fragmentation of protected areas and increase connectivity among the natural reserves. This will also stimulate these productive systems to shift from a source of carbon to sink and thus help mitigating climate change impacts. (3) Many sensitive areas are located in the border between Andean countries. Therefore it is important to include key binational reserves where climate change impacts are likely to be severe. This consideration calls for a regional conservation agenda were political platforms such as the Andean Community (CAN) can be of much help to foster conservation actions that are beyond country governance.

### 4.3 Evaluating the biome modelling approach and outlook

The uncertainty analysis of the biome modelling shows no overlap in confidence intervals between probability of occurrence of the most probable biome and another biome for more than 90% of the modelled area. Together with an overall accuracy of 89%, this suggests that our approach to model Tropical Andean biomes is robust. Areas with overlaps between the confidence intervals may be caused by the coarse resolution and interpretation errors of the map, but they can also represent ecotones between biomes.

Another potential issue of climate change impact assessments is the need to project outside the current climate envelope, which poses fundamental issues of model reliability [65]. However, the large variability of current climates in the tropical Andes and its buffer zone results in only a very small fraction of non-analogue climate combinations only for the 2040–2069 period, which again should make the modelling exercise relatively robust. One potential pathway for improvement is to account for the effect of the combined variables in the analysis of the observed envelopes. Our approach did not do this because of the large number of variables included. This may result, for example, in small deviation of the observed values being assigned as new climates, which could overestimate the non-analogue climates. An alternative approach may be to identify non-analogue climates by areas where the model predicts low probabilities for all biomes, or where it is hard to differentiate between the most probable and other biomes. Further research is needed in this matter.

Finally, more solid scenarios should ideally incorporate a dynamic model of land use change. The absence of good quality data such as past land use trajectories for the whole region and updated detailed land cover maps for some of the countries is currently a major limiting factor. Additionally the interactions between the vegetation and water cycle should also be taken into account but this is currently limited not only by the lack of a conceptual model for the Tropical Andes but for the absence of higher resolution climatic layers or information on the climatic interannual variability. A better understanding of biological processes and limiting factors on the Andes such as dispersal and seed establishment is also needed.

## Conclusions

According to our projections, the Tropical Andes will not respond homogeneously to climate change. Different conservation and adaptation measures should therefore be designed accordingly. Some biomes are projected to experience an upslope displacement of both the upper and lower boundaries, while others are projected to expand downslope. The projected upslope displacement is supported by palaeoecological evidence from post-glacial time; however, future temperature anomalies are projected to be higher and result from faster rates of change than in the past. Biome displacement will need species to have faster migration rates than present. Additionally, human land use has already transformed important areas of the Andean landscape, which has a strong effect on biome resilience. However, the interaction between climate change and land use change is further complicated. Since we assumed a static land use scenario, this impact is underestimated given that future land use change is expected to increase. The downslope expansion projected for the dry biomes (seasonally dry tropical montane forest, montane shrubland, xeric pre-puna) may result from changes in the water balance but this needs further study in the Andes.

In contrast with other studies at species level, large areas of the Tropical Andes are projected to remain stable (from 74.8% to 83.1%). However, several biomes are projected to lose more than 30% of their current area. Vulnerable areas include the biomes which are currently already most threatened (glaciers and cryoturbated areas, paramo and evergreen montane forest) but also specific areas under stress due to changes in physiognomy or humidity levels. The identification of these areas including different climatic models accounts for the uncertainty of future climate projections. The inclusion of the uncertainty analysis by means of a GCM model ensemble has also implications for management decisions such the establishment of protected areas in regions with less uncertainty.

Future work should focus on improving the biome modelling, which is currently limited by data availability and lack of knowledge of specific processes. Despite its simplifications, the good overall adjustment of our model shows that it is possible to assess biome distribution changes at fine resolution to inform decision-making. Additionally, our methodology can be applied to other tropical mountain ecosystems as well.

## Supporting Information

Figure S1 **Maps representing uncertainty analysis of the biome model and non-analogue climates.** A) Map showing the number of overlaps between the confidence interval of the most probable biome and other biomes for the present. B) Map showing the richness of non-analogue climates for the future under scenario A2 2040–2069 based on the summed occurrence of all variables exceeding the range of calibrated data for all models.
(TIF)

Figure S2 **Density functions of the selected biome probability and standard deviation, according to the number of overlaps between the confidence interval of the selected biome and another biome or biomes.**
(PNG)

Figure S3 **Median change in the area of potential biomes versus remnant biomes under A2 scenario.** In dark grey the lost areas (the biome will be replaced by another biome), in grey stable areas (areas that remained unchanged) and in light grey new or emerging areas (the biome is projected to occur in the future but not in the present). Bars represent the minimum and maximum values of all models.
(EPS)

Figure S4 **Median change in the area of potential biomes for each model under A1B scenario, 2010–2039.**
(EPS)

Figure S5 **Median change in the area of potential biomes for each model under A1B scenario, 2040–2069.**
(EPS)

Figure S6 **Median change in the area of remnant biomes for each model under A1B scenario, 2010–2039.**
(EPS)

Figure S7 **Median change in the area of remnant biomes for each model under A1B scenario, 2040–2069.**
(EPS)

Table S1 **Variables used for each biome model.**
(DOC)

Table S2 **Median relative area changes between future and present for potential and remnant biomes (A1B 2010–2039 and A2 2040–2069).**
(DOC)

Table S3 **Conversion matrix from present biomes to future projected biomes for scenario A2 2040–2069.**
(DOC)
