# Supplementary figures and images for: Diverging Responses of Tropical Andean Biomes under Future Climate Conditions
Source: PLoS One. 2013 May 7;8(5):e63634. doi: 10.1371/journal.pone.0063634 (PMC3646809; doi:10.1371/journal.pone.0063634)

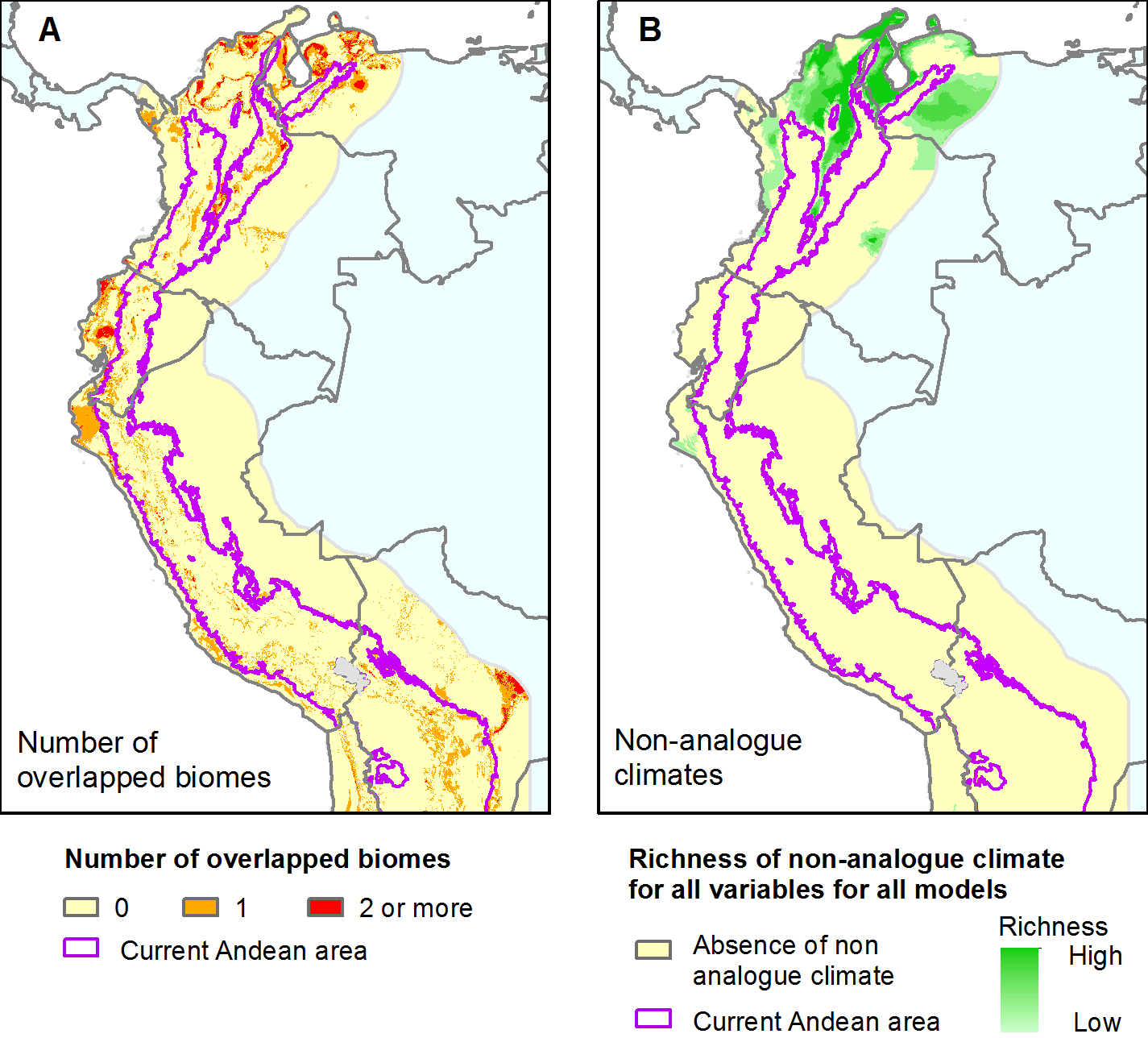

Supplement: Figure S1 — Maps representing uncertainty analysis of the biome model and non-analogue climates. A) Map showing the number of overlaps between the confidence interval of the most probable biome and other biomes for the present. B) Map showing the richness of non-analogue climates for the future under scenario A2 2040–2069 based on the summed occurrence of all variables exceeding the range of calibrated data for all models. (TIF) [file pone.0063634.s001.tif]

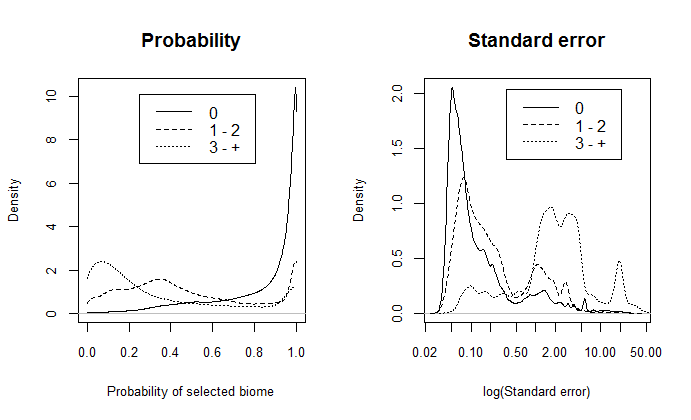

Supplement: Figure S2 — Density functions of the selected biome probability and standard deviation, according to the number of overlaps between the confidence interval of the selected biome and another biome or biomes. (PNG) [file pone.0063634.s002.png]
