# Supplementary material for: Diverging Responses of Tropical Andean Biomes under Future Climate Conditions
Source: PLoS One. 2013 May 7;8(5):e63634. doi: 10.1371/journal.pone.0063634 (PMC3646809; doi:10.1371/journal.pone.0063634)
Supplement: Table S1 — Variables used for each biome model. (DOC) [file pone.0063634.s008.doc]

**Table S1** Variables used for each biome model.

| Variables | GC | P | HP | XP | MS | EMF | SDTF1 | SDTF2 | PP |
| --- | --- | --- | --- | --- | --- | --- | --- | --- | --- |
| Intercept | (-) * | (-) *** | (-) *** | (-) *** | (-) *** | (-) *** | (-) *** | (-) *** | (-) *** |
| Annual mean T | (-) *** | (-) *** | (-) *** | (-) ** | (-) *** | (+) *** | (+) *** | (-) *** | (-) *** |
| Mean monthly range T | (+) * | (+) *** | (+) *** | (-) *** |  | (-) *** | (-) * | (+) *** | (+) *** |
| Annual pp ^1^ |  | (+) *** | (+) *** | (-) *** | (+) *** | (+) *** |  |  | (+) *** |
| pp of driest month ^1^ |  | (+) *** | (+) *** | (+) *** | (-) *** | (+) ** |  | (+) *** | (+) *** |
| pp of coldest quarter ^1^ | (+) ** | (+) *** | (-) *** | (+) *** | (-) *** | (-) *** | (+) *** | (+) *** | (+) *** |
| pp of warmest quarter ^1^ | (+) ** | (+) . | (-) *** | (+) *** | (+) *** | (-) ** | (-) *** | (-) *** | (-) ** |
| Coefficient of variation of pp |  | (+) | (-) | (+) *** | (+) * | (-) *** | (+) *** | (-) * | (+) * |
| Ombrothermic index ^1^ | (-) *** | (-) *** | (-) ** | (+) *** | (-) *** |  | (+) *** | (+) *** | (-) *** |
| Ombrothermic index of the driest bimonth ^1^ |  | (-) *** | (-) *** | (+) *** | (+) *** | (+) *** | (-) *** | (-) *** | (-) *** |
| Convergence index | (+) *** | (+) ** | (+) ** | (+) *** | (-) . |  |  |  | (+) * |
| Terrain ruggedness index ^1^ | (+) * | (+) . | (+) *** | (-) *** | (+) *** | (+) *** | (+) *** | (+) *** | (+) *** |
| Slope ^1^ |  |  | (-) ** | (+) *** | (+) * | (-) * |  | (-) * |  |
| Annual mean T (quadratic term) | (-) ** | (+) *** | (-) *** | (-) ** | (+) ** | (-) *** | (-) *** | (+) |  |
| Mean monthly range T (quadratic term) | (-) . | (-) *** | (-) *** |  | (-) *** | (+) *** | (+) ** | (-) *** | (-) *** |
| Annual pp (quadratic term) ^1^ | (+) * | (-) *** | (-) *** | (+) *** | (-) *** | (-) *** | (-) . | (+) *** | (-) *** |
| pp of Driest Month (quadratic term) ^1^ |  | (-) *** | (-) *** | (-) *** | (+) *** | (-) * | (-) *** | (-) *** |  |
| pp of coldest quarter (quadratic term) ^1^ | (-) ** | (-) *** | (+) * | (-) *** | (+) *** | (+) *** | (-) *** | (-) *** | (-) ** |
| pp of warmest quarter (quadratic term) ^1^ | (-) ** | (-) * | (+) *** | (-) *** | (-) *** | (+) *** | (+) *** | (+) *** | (+) *** |
| Coefficient of variation of pp (quadratic term) | (-) *** | (-) . | (-) ** | (-) *** | (-) ** | (+) | (-) *** | (+) *** | (-) |
| Ombrothermic index (quadratic term) ^1^ | (+) *** | (+) *** | (+) | (-) *** | (-) *** | (+) *** | (-) *** | (-) *** | (+) *** |
| Ombrothermic index of the driest bimonth (quadratic term) ^1^ | (+) *** | (+) *** | (+) *** | (-) . | (-) *** | (-) *** | (+) *** | (+) *** |  |
| Convergence index (quadratic term) | (-) *** | (-) . | (-) . |  | (+) ** | (-) *** | (+) . | (+) *** |  |
| Terrain ruggedness index (quadratic term) ^1^ | (-) *** | (-) * |  | (+) *** | (-) *** | (-) *** | (-) *** | (-) *** | (-) *** |
| slope (quadratic term) ^1^ | (+) *** |  | (+) *** | (-) *** | (-) * |  |  | (+) ** | (+) *** |
| Residual deviance | 1814.6 | 3235 | 15310 | 13461 | 15257 | 30683 | 25417 | 19391 | 3875.1 |
| df residual deviance | 124204 | 124200 | 124199 | 124200 | 124199 | 124201 | 124203 | 124200 | 124203 |
| AUC | 0.999 | 0.999 | 0.994 | 0.994 | 0.965 | 0.971 | 0.939 | 0.964 | 0.997 |
| ^1^ Variables that were log-transformed , (+) indicates a positive relations while (-) indicates a negative relation. | | | | | |  |  |  |  |
| Areas of Seasonal montane forest (SDTF1) and Xeric montane forest (SDTF2) were merged to define SDTF as this gave the best fit for this biome. | | | | | | | | | |
