# Supplementary material for: Diverging Responses of Tropical Andean Biomes under Future Climate Conditions
Source: PLoS One. 2013 May 7;8(5):e63634. doi: 10.1371/journal.pone.0063634 (PMC3646809; doi:10.1371/journal.pone.0063634)
Supplement: Table S2 — Median relative area changes between future and present for potential and remnant biomes (A1B 2010–2039 and A2 2040–2069). (DOC) [file pone.0063634.s009.doc]

**Table S2** Median relative area changes between future and present for potential and remnant biomes (A1B 2010-2039 and A2 2040-2069).

| Biome | type | A1B 2010-2039 | | | A2 2040-2069 | | |
| --- | --- | --- | --- | --- | --- | --- | --- |
|  |  | % lost areas | % stable areas | % emerging areas | % lost areas | % stable areas | % emerging areas |
| Glaciers and cryoturbated areas | Potential | 57.7 | 42.3 | 0.1 | 82.5 | 17.5 | 0.0 |
|  |  | (49 - 63.9) | (36.1 - 51) | (0 - 0.4) | (68.3 - 86.3) | (13.7 - 31.7) | (0 - 0.1) |
|  | Remnant | 57.7 | 42.3 | 0.1 | 82.5 | 17.5 | 0.0 |
|  |  | (49 - 63.9) | (36.1 - 51) | (0 - 0.4) | (68.3 - 86.3) | (13.7 - 31.7) | (0 - 0.1) |
| Paramo | Potential | 31.4 | 68.6 | 0.8 | 48.5 | 51.5 | 0.9 |
|  |  | (17.3 - 38.8) | (61.2 - 82.7) | (0.3 - 1.3) | (29.3 - 54.8) | (45.2 - 70.7) | (0.3 - 1.4) |
|  | Remnant | 25.0 | 75.0 | 1.0 | 40.9 | 59.1 | 1.2 |
|  |  | (11.9 - 35.6) | (64.4 - 88.1) | (0.3 - 1.6) | (24 - 47.3) | (52.7 - 76) | (0.4 - 1.7) |
| Humid puna | Potential | 6.6 | 93.4 | 2.9 | 11.9 | 88.1 | 5.2 |
|  |  | (3.8 - 9.3) | (90.7 - 96.2) | (0.9 - 11.6) | (6.7 - 21.4) | (78.6 - 93.3) | (1.1 - 7.6) |
|  | Remnant | 6.0 | 94.0 | 3.1 | 10.6 | 89.4 | 5.6 |
|  |  | (3.4 - 8.6) | (91.4 - 96.6) | (1 - 12) | (6.1 - 20) | (80 - 93.9) | (1.2 - 8.2) |
| Xeric puna | Potential | 8.3 | 91.7 | 4.5 | 10.9 | 89.1 | 5.0 |
|  |  | (3.6 - 14.9) | (85.1 - 96.4) | (2.6 - 8.2) | (6 - 15.6) | (84.4 - 94) | (3.2 - 17.3) |
|  | Remnant | 8.2 | 91.8 | 4.3 | 10.7 | 89.3 | 4.8 |
|  |  | (3.6 - 14.6) | (85.4 - 96.4) | (2.6 - 8) | (5.9 - 15.5) | (84.5 - 94.1) | (3.2 - 16.3) |
| Evergreen montane forest | Potential | 18.0 | 82.0 | 6.5 | 29.1 | 70.9 | 10.3 |
|  |  | (12.8 - 24.5) | (75.5 - 87.2) | (3.3 - 9.2) | (24.4 - 33.9) | (66.1 - 75.6) | (5 - 17.4) |
|  | Remnant | 20.0 | 80.0 | 7.0 | 30.0 | 70.0 | 11.5 |
|  |  | (12.7 - 22.3) | (77.7 - 87.3) | (3.3 - 11.2) | (23.9 - 36.6) | (63.4 - 76.1) | (5.5 - 20.8) |
| Seasonally dry tropical montane forest | Potential | 14.7 | 85.3 | 35.7 | 16.0 | 84.0 | 63.2 |
|  |  | (7.6 - 23) | (77 - 92.4) | (24.3 - 52.5) | (13.1 - 23.9) | (76.1 - 86.9) | (42.6 - 82.6) |
|  | Remnant | 14.6 | 85.4 | 35.1 | 15.8 | 84.2 | 62.0 |
|  |  | (7.5 - 22.2) | (77.8 - 92.5) | (23.5 - 52.1) | (12.6 - 23) | (77 - 87.4) | (42.7 - 81.6) |
| Montante shrublands | Potential | 24.3 | 75.7 | 31.3 | 40.7 | 59.3 | 59.0 |
|  |  | (13.4 - 38.7) | (61.3 - 86.6) | (19.4 - 51.5) | (18.7 - 54.4) | (45.6 - 81.3) | (40.7 - 122.2) |
|  | Remnant | 22.6 | 77.4 | 25.4 | 39.4 | 60.6 | 48.7 |
|  |  | (11.7 - 38.2) | (61.8 - 88.3) | (15.6 - 47.6) | (15.5 - 55) | (45 - 84.5) | (32.4 - 125.8) |
| Xeric pre-puna | Potential | 7.1 | 92.9 | 15.1 | 6.5 | 93.5 | 20.7 |
|  |  | (3.3 - 11.2) | (88.8 - 96.7) | (13.2 - 22.8) | (4 - 14.9) | (85.1 - 96) | (15.3 - 32.5) |
|  | Remnant | 7.1 | 92.9 | 15.0 | 6.4 | 93.6 | 20.2 |
|  |  | (3.3 - 10.9) | (89.1 - 96.7) | (12.8 - 23.1) | (4 - 14.5) | (85.5 - 96) | (15.2 - 32.9) |

Calculations for the potential biomes and the remnant biomes are shown with the range of values in brackets.
