# Supplementary material for: Diverging Responses of Tropical Andean Biomes under Future Climate Conditions
Source: PLoS One. 2013 May 7;8(5):e63634. doi: 10.1371/journal.pone.0063634 (PMC3646809; doi:10.1371/journal.pone.0063634)
Supplement: Table S3 — Conversion matrix from present biomes to future projected biomes for scenario A2 2040–2069. (DOC) [file pone.0063634.s010.doc]

**Table S3.** Conversion matrix from present biomes to future projected biomes for scenario A2 2040-2069.

| Present/  Future | GC | P | HP | XP | EMF | SDTF | MS | PP | NAB |
| --- | --- | --- | --- | --- | --- | --- | --- | --- | --- |
| GC | 17.5 | 0.0 | 32.5 | 48.3 | 0.0 | 0.0 | 0.0 | 0.0 | 0.4 |
|  | (13.7 - 31.7) | (0 - 0) | (10.7 - 42.3) | (37.8 - 72.9) | (0 - 0) | (0 - 0) | (0 - 0) | (0 - 0) | (0 - 2.7) |
| P | 0.0 | 51.5 | 0.2 | 0.0 | 44.4 | 0.2 | 2.9 | 0.0 | 0.0 |
|  | (0 - 0) | (45.2 - 70.7) | (0 - 3.3) | (0 - 0) | (23.2 - 52.4) | (0 - 2.5) | (1.3 - 4.3) | (0 - 0) | (0 - 1.6) |
| HP | 0.0 | 0.1 | 88.0 | 1.4 | 5.2 | 2.3 | 0.8 | 0.2 | 0.0 |
|  | (0 - 0) | (0 - 0.2) | (78.6 - 93.3) | (0.1 - 11.9) | (1.9 - 9.3) | (1.2 - 5.8) | (0.3 - 1.5) | (0.1 - 0.5) | (0 - 0.1) |
| XP | 0.0 | 0.0 | 2.7 | 89.1 | 0.0 | 4.3 | 1.1 | 1.5 | 0.6 |
|  | (0 - 0) | (0 - 0) | (0 - 4.7) | (84.4 - 94) | (0 - 0) | (3.1 - 7.5) | (0.4 - 3.4) | (1.1 - 1.9) | (0.4 - 0.8) |
| EMF | 0.0 | 0.0 | 0.0 | 0.0 | 70.9 | 8.3 | 1.6 | 0.0 | 19.2 |
|  | (0 - 0) | (0 - 0.1) | (0 - 0) | (0 - 0) | (66.1 - 75.6) | (3.7 - 12.1) | (0.3 - 2.4) | (0 - 0) | (13.5 - 24.8) |
| SDTF | 0.0 | 0.0 | 0.0 | 0.8 | 0.3 | 84.0 | 1.8 | 0.9 | 11.1 |
|  | (0 - 0) | (0 - 0) | (0 - 0) | (0.1 - 1.4) | (0.1 - 2.4) | (76.1 - 86.9) | (0.6 - 3.8) | (0.2 - 1.2) | (8.7 - 19.8) |
| MS | 0.0 | 0.0 | 0.0 | 0.0 | 0.4 | 32.9 | 59.3 | 0.6 | 5.7 |
|  | (0 - 0) | (0 - 0) | (0 - 0) | (0 - 0) | (0.1 - 1.9) | (10.4 - 46.1) | (45.6 – 81.3) | (0 - 2.5) | (4.7 - 7.6) |
| PP | 0.0 | 0.0 | 0.0 | 0.1 | 0.0 | 1.4 | 1.1 | 93.4 | 3.1 |
|  | (0 - 0) | (0 - 0.1) | (0 - 0.3) | (0 - 0.8) | (0 - 0) | (0.5 - 5.1) | (0 - 5.8) | (85.1 - 96) | (2.5 - 7.9) |
| NAB | 0.0 | 0.0 | 0.0 | 0.0 | 0.0 | 2.9 | 0.7 | 0.2 | 96.1 |
|  | (0 - 0) | (0 - 0) | (0 - 0) | (0 - 0) | (0 - 0.5) | (1.8 - 4.5) | (0.1 - 2.1) | (0.1 - 0.7) | (92.7 - 97.3) |

Median change in area (%) of all models, for scenario A2 2040-2069, between baseline (rows) and potential future biomes (columns). Minimum and maximum values of all models are shown in brackets. GC=glaciers and cryoturbated areas, P=paramo, HP=humid puna, XP=xeric puna, EMF=evergreen montane forest, SDTF=seasonally dry tropical montane forest, MS=montane shrubland, PP=xeric pre-puna, NAB=non-Andean biome.
